# Synthesis of Zn–Fe double metal cyanide complexes with imidazolium-based ionic liquid cocatalysts *via* ball milling for copolymerization of CO_2_ and propylene oxide

**DOI:** 10.1039/c7ra12528c

**Published:** 2018-02-09

**Authors:** Jia Shi, Zaifeng Shi, Huiqiong Yan, Xianghui Wang, Xiaopeng Zhang, Qiang Lin, Linhua Zhu

**Affiliations:** College of Chemistry and Chemical Engineering, Hainan Normal University Haikou Hainan 571158 P. R. China linqianggroup@163.com zhulinhua1981@163.com +86-0898-65889422 +86-0898-65889422; Key Laboratory of Pollution Control of Hainan Province, Hainan Normal University Haikou Hainan 571158 P. R. China; Key Laboratory of Tropical Medicinal Plant Chemistry of Ministry Education, Hainan Normal University Haikou Hainan 571158 P. R. China

## Abstract

In this work, Zn–Fe double metal cyanide (DMC) catalysts were successfully synthesized *via* clean and efficient ball milling. Imidazolium-based ionic liquids as cocatalysts were incorporated into the structure of the DMC catalysts during the grinding process. The modified Zn–Fe DMC catalysts were effective for the alternating copolymerization of carbon dioxide and propylene oxide under controlled reaction conditions. The properties and structures of the Zn–Fe DMC catalysts and the resulting polymers were characterized by Fourier transform infrared spectroscopy, scanning electron microscopy, X-ray diffraction, elemental analysis, ^1^H and ^13^C NMR spectroscopy, gel permeation chromatography, and thermogravimetric analysis. The results indicate that the Zn–Fe DMC catalysts exhibit higher thermal stability compared to the DMC catalysts without imidazolium-based ionic liquids (DMC-Blank). We determined that the introduction of a small amount of imidazolium-based ionic liquids can increase the carbonate content of the poly(propylene carbonate) (PPC) copolymer in the range of 18.48–29.00%. The turnover numbers of PPC were ∼4.40. In addition, the measured number-average relative molecular mass was in the range of 2.96 × 10^3^–4.98 × 10^3^ with a narrow polydispersity index of 1.00–1.08.

## Abstract 2

Zn–Fe DMC complexes with imidazolium-based ionic liquid cocatalysts were prepared by a clean and efficient ball milling process for the copolymerization of CO_2_ and propylene oxide.

## Graphical abstract

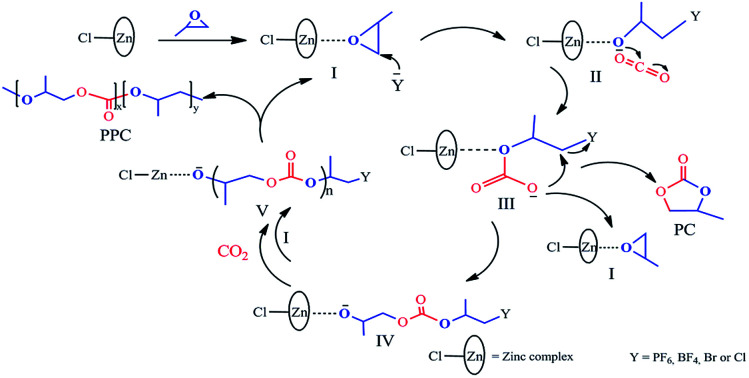

## Introduction

Carbon dioxide (CO_2_) is an ideal synthetic C1 resource because it is abundant, renewable, inexpensive, non-toxic and nonflammable.^1–5^ The utilization of CO_2_ for the industrial production of aliphatic polycarbonates has attracted significant attention in recent years.^6,7^ CO_2_ can be effectively fixed in the copolymerization reaction with epoxides, leading to the synthesis of polycarbonates. Polycarbonates possess outstanding properties: they are highly transparent, durable, biodegradable, and light. As such, they have useful applications in the healthcare, food, and the automotive industries.^8–11^

It is generally accepted that effective catalysts are critical for the successful synthesis of propylene carbonates from CO_2_ and epoxides. Since the initial discovery by Inoue and coworkers of the ZnEt_2_ and water catalyst for the reaction of CO_2_ and epoxides,^12,13^ researchers have developed a wide variety of catalytic systems for the synthesis of polycarbonates including zinc carboxylates,^14^ Salen metal compounds,^15^ β-diiminate zinc derivatives,^16^ organic bases,^17^ metalloporphyrins,^18^ a rare-earth ternary catalyst,^19^ organic ammonium halogenides and others.^20–23^ Among these, double metal cyanide (DMC) catalysts display excellent catalytic activity for the copolymerization of CO_2_ and epoxides *via* several different reaction mechanisms.^24–27^ However, the complicated reaction processes, harsh synthesis conditions, poor activity, and unsatisfactory thermal stability of these catalysts are major drawbacks that urgently need to be addressed. In this regard, the simple and efficient process of mechanochemistry has proven to be effective in activating the copolymerization of CO_2_ and epoxides.^28–31^

Catalysts can be synthesized for polycarbonates using a metal complex. They can also be used together with a cocatalyst to enhance their performance in a binary catalyst system. Several different binary catalyst systems have been synthesized for polymerization with good performance.^32–34^ Ionic liquids (IL) as cocatalysts have unique characteristics which include environmental benignity, thermal and chemical stability, and low volatility compared to conventional organic and inorganic solvents.^35–37^

**Scheme 1:**
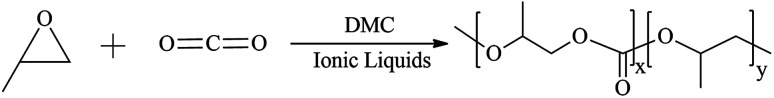
Reaction of CO_2_ and propylene oxide.

In this work, binary catalyst systems of Zn–Fe DMC catalysts were synthesized by the addition of cocatalysts of imidazolium-based ionic liquids (IL) during ball milling. We investigated the properties and structures of the Zn–Fe DMC complexes and the polymers which resulted from the copolymerization of CO_2_ and propylene oxide (PO) (Scheme 1). The copolymerization of CO_2_ and PO catalyzed by Zn–Fe DMC complexes with imidazolium-based ionic liquids as cocatalysts, was investigated in this report.

## Experimental

### Materials

Carbon dioxide (CO_2_) (purity > 99.9%) and propylene oxide (PO) (≥98%) were used. The chemicals potassium hexacyanoferrate(iii) [K_3_Fe(CN)_6_], zinc chloride (ZnCl_2_), 1-butyl-3-methylimidazolium hexafluorophosphate (BMIMPF_6_), 1-butyl-3-methylimidazolium tetrafluoroborate (BMIMBF_4_), 1-butyl-3-methylimidazolium bromide (BMIMBr), 1-butyl-3-methylimidazolium chloride (BMIMCl), *tert*-butyl alcohol (*t*-BuOH), and trichloromethane (CHCl_3_) were of analytical purity and used directly without further purification.

### Synthesis of Zn–Fe DMC catalysts

The synthesis of the Zn–Fe DMC catalysts was carried out with a planetary ball mill. A mixture of ZnCl_2_ and K_3_Fe(CN)_6_ (molar ratio of 10 : 1) with 2 mL of *t*-BuOH was placed separately into five stainless steel vessels. BMIMPF_6_, BMIMBF_4_, BMIMBr, and BMIMCl were then added to vessels 2, 3, 4, and 5, respectively, but not to vessel 1. Vessels 1–5 were labelled DMC-Blank, DMC-PF_6_, DMC-BF_4_, DMC-Br and DMC-Cl respectively. Subsequently, the mixture was ground at 50 Hz for 10 min. The resulting products were first thoroughly washed with a mixed solution of deionized water and *t*-BuOH (volume ratio of 1 : 1) to remove excess reactants, then filtered and dried under vacuum at 55 °C.

### Copolymerization of CO_2_ and PO

Copolymerization of CO_2_ and PO was performed in a 500 mL stainless steel autoclave equipped with a magnetic stirrer, heating water bath, and a check valve. A total of 0.5 g of dried DMC was added to the autoclave, followed by the addition of 30 mL of PO under vacuum conditions. The reactor was then heated to 60 °C and pressurized at 3 MPa with CO_2_. After 24 h, the autoclave was cooled to room temperature, slowly depressurized, and opened. The resulting products were dissolved in trichloromethane, stirred for 15 min, poured into water, isolated, and dried under vacuum at 55 °C for several hours prior to characterization.

### Characterization

The DMC catalysts were characterized using a scanning electron microscope (Model: JEM-7100F), X-ray diffractometer (Bruker AXS/D8), thermogravimetric analyzer at a heating rate of 10 °C min^−1^ under O_2_ atmosphere in a high throughput mode (SDT Q600), elemental analyzer (Vario EL Cube), and Fourier transform infrared (FT-IR) spectrometer using KBr tablets (6700/Thermo Fisher Scientific). NMR spectroscopic analyses of the products were performed using a Bruker NMR spectrometer (Model: Bruker AV 400 MHz) with ^1^H and ^13^C probes, and CDCl_3_ as the solvent. The *M*_n_ and polydispersity indices (PDI) of the polymer products were estimated using a gel permeation chromatography system.

## Results and discussion

### Characterization of DMC catalysts

**Fig. 1:**
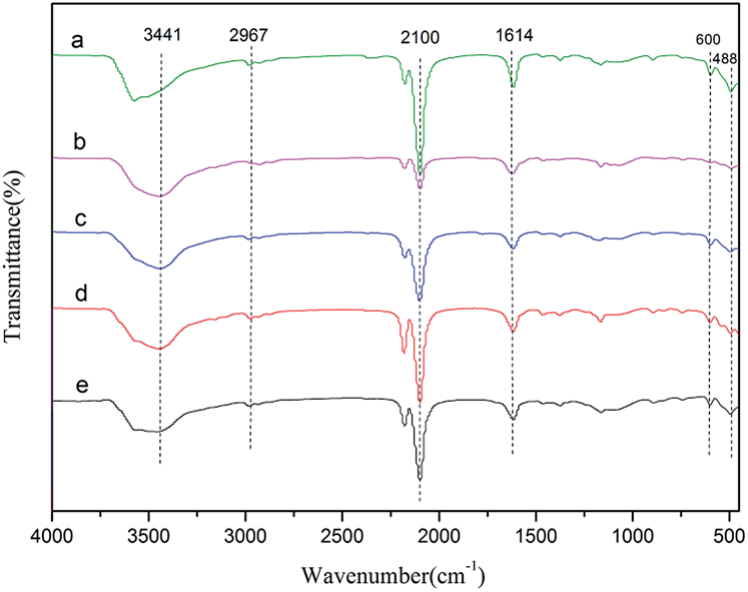
FTIR spectra of DMC-Blank (a), DMC-PF_6_ (b), DMC-BF_4_ (c), DMC-Br (d) and DMC-Cl (e).

FT-IR spectra of the Zn–Fe DMC catalysts prepared by grinding with various imidazolium-based ionic liquids are shown in Fig. 1. All samples exhibited characteristic absorption peaks which corresponded to those of the Zn–Fe DMC catalysts prepared without ionic liquids (DMC-Blank). For example, the peaks of DMC-PF_6_ at ∼2100 cm^−1^ were assigned to the C≡N stretching mode of the Zn^2+^–CN–Fe^3+^ unit. An absorption peak at 1619 cm^−1^ could also be clearly observed in the spectra. This stretching vibration corresponded to the weakened C≡N bond in the Zn^2+^–CN–Fe^3+^ unit, which resembled the C=N bond. The weakening of the C≡N bond was ascribed to the coordination of Zn^2+^ with the C≡N group. Due to the presence of the complexing agent *tert*-BuOH, the stretching vibration absorption peaks of the hydroxyl groups appears at 3441 cm^−1^. In contrast, the peak at 2948 cm^−1^ was attributed to the C–H stretching vibration absorption and the Fe–CN flexural vibration absorption peaks are located at 600 and 482 cm^−1^. The C=N bond was shifted to higher frequencies compared with DMC-Blank. This indicates that CN^−^ acts as a σ-donor by donating electrons to Fe^3+^, and as a π-electron donor by chelating to Zn^2+^.^38^ These FT-IR results also confirm the existence of typical functional groups in the Zn–Fe DMC catalysts.

**Fig. 2:**
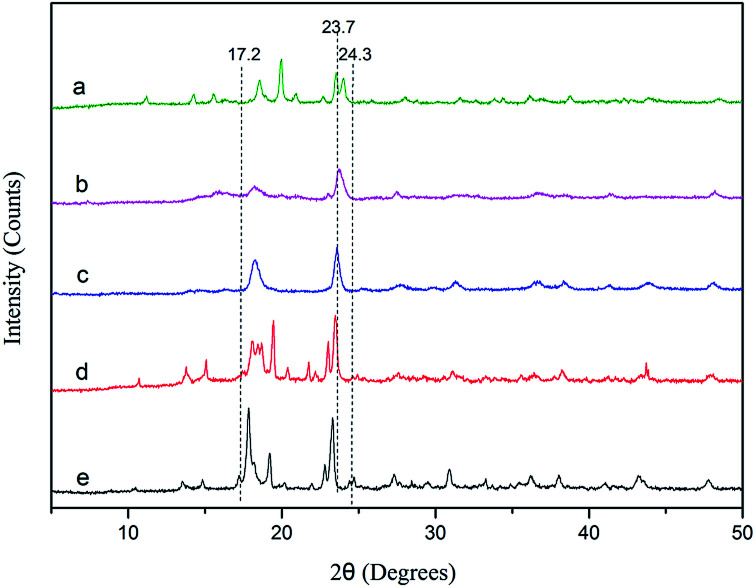
XRD patterns of DMC-Blank (a), DMC-PF_6_ (b), DMC-BF_4_ (c), DMC-Br (d) and DMC-Cl (e).

The X-ray diffraction (XRD) patterns of the DMC catalysts are depicted in Fig. 2. There are no obvious differences in the crystal patterns of DMC-PF_6_, DMC-BF_4_, DMC-Br, DMC-Cl, and DMC-Blank. The observed peaks of the Zn–Fe DMC catalysts are in the range of 2*θ* = 17.2–24.3° and are of low intensities. However, the reflections at approximately 23.7° (2*θ*) of the DMC catalyst exhibited a pronounced intensity, which is characteristic of a monoclinic phase with lower phase purity.^39,40^ It was inferred that the DMC catalysts prepared by ball milling showed high catalytic activity because of their lower crystallinity. Thus, the PO ring could be easily opened, which allows for better penetration of the reactants into the catalyst structure.

**Fig. 3:**
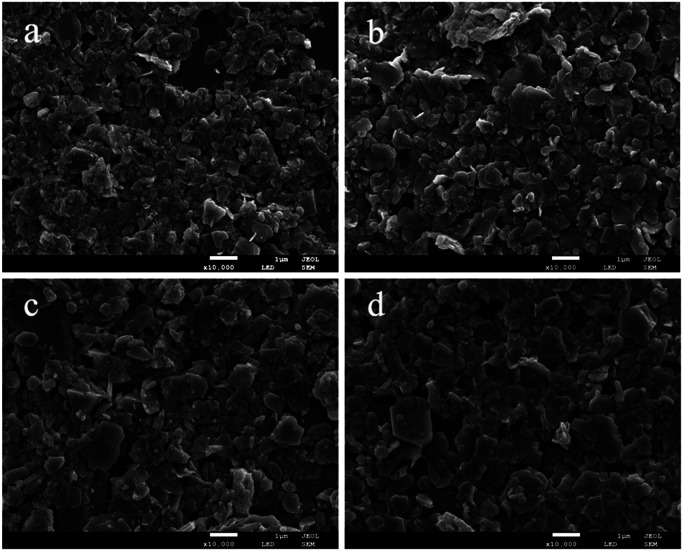
SEM images of the DMC-PF_6_ (a), DMC-BF_4_ (b), DMC-Br (c) and DMC-Cl (d).

An effort was made to examine the morphology and surface shape of the synthesized DMC catalysts using scanning electron microscopy (SEM). As evident from Fig. 3, the SEM images showed that the minimum dimensions of the particles were up to a few hundred nanometers. The physical appearance of the catalysts confirmed the presence of sheet structures and overlapping of the particles with each other. The fragmentation of the Zn–Fe DMC catalysts allowed for an easier introduction of the IL cocatalysts into the catalysts structure. This also resulted in the formation of a high surface area and more activity points for the reactants by the grinding method. As such, a high catalytic activity in the polymerization of CO_2_ and PO is observed.^41^

The elemental composition of the as-prepared Zn–Fe DMC catalysts with various imidazolium-based ionic liquids was determined. The results of these analyses are shown in Table 1. The N and C contents of DMC-PF_6_, DMC-BF_4_, DMC-Br, and DMC-Cl are relatively lower than that in DMC-Blank. This result indicate that the ionic liquids are incorporated into the catalysts according to the N and C content, which also shows Zn–Fe DMC catalysts synthesized by ball milling.

**Table 1:** Elemental composition of the obtained Zn–Fe DMC catalysts in terms of mass fractions

Entry	Catalyst	IL	C (wt%)	H (wt%)	N (wt%)
1	DMC-Blank	—	21.99	3.92	16.63
2	DMC-PF_6_	BMIMPF_6_	13.69	1.95	12.24
3	DMC-BF_4_	BMIMBF_4_	9.21	2.69	8.41
4	DMC-Br	BMIMBr	19.18	3.13	13.96
5	DMC-Cl	BMIMCl	17.60	3.68	13.35

**Fig. 4:**
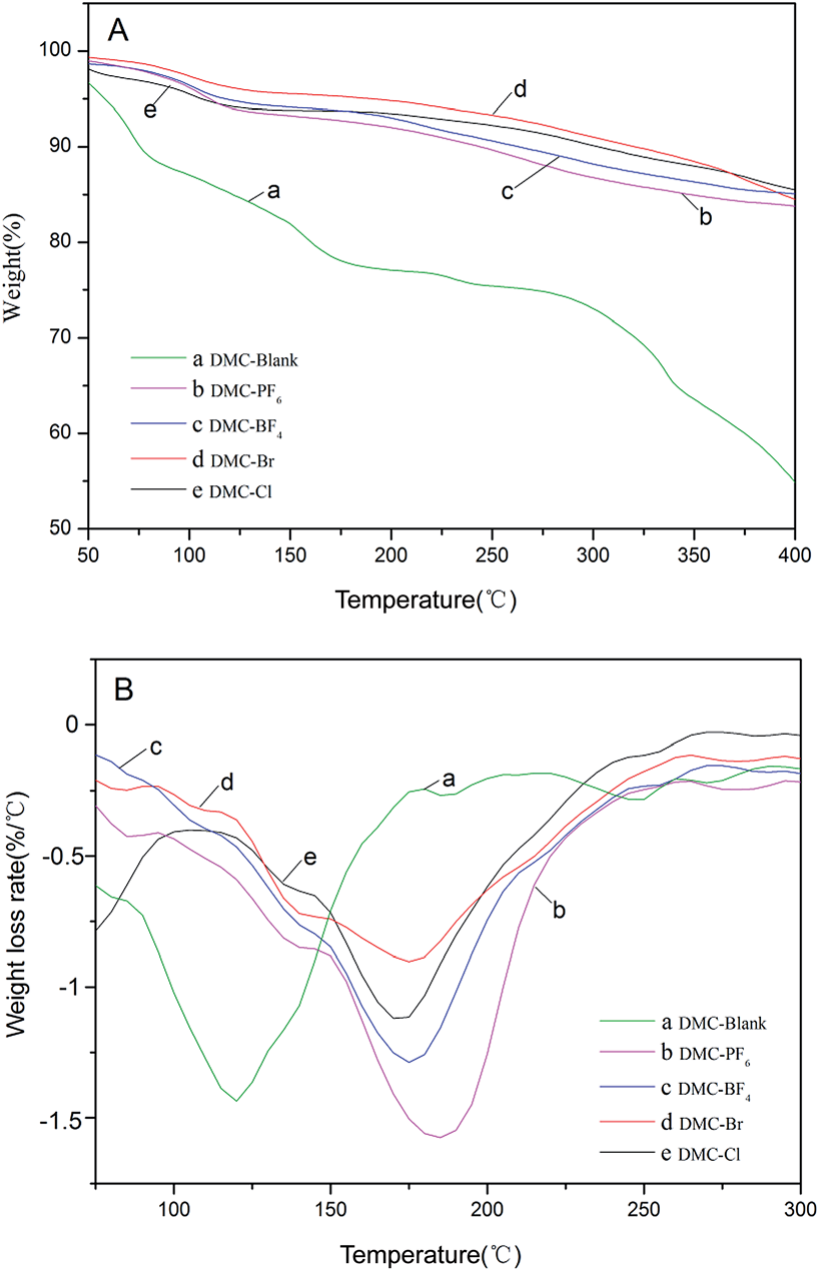
TGA (A) and DTG (B) profiles of DMC-Blank (a), DMC-PF_6_ (b), DMC-BF_4_ (c), DMC-Br (d) and DMC-Cl (e).

The thermal stability of the DMC catalysts is an important consideration in the determination of the temperature for activation. The activation temperatures for the synthesized catalysts were investigated by means of thermogravimetric analysis (TGA) and derivative thermogravimetry (DTG) analysis in a N_2_ atmosphere in a high throughput mode (Table 2 and Fig. 4). The decomposition of DMC catalysts resulted in an initial slight weight loss below 120 °C, which was possibly due to the removal of H_2_O and *t*-BuOH from the surface of the catalysts, followed by the loss of IL moieties and cyanide ligands.^42^ Furthermore, a second significant mass loss was observed in the range of 150–220 °C for the DMC-Blank. This indicated that the catalyst was thermally stable up to ∼250 °C. A total weight loss of 30% was observed for the DMC catalysts with ILs including DMC-PF_6_ (184 °C) > DMC-BF_4_ (176 °C) > DMC-Br (175 °C) > DMC-Cl (172 °C) > DMC-Blank (119 °C). Thus, DMC-PF_6_ is expected to exhibit good stability for catalytic reactions performed at moderately high temperatures.

**Table 2:** Thermal properties of the DMC catalysts

Entry	Catalyst	*T*_−5%_/°C	*T*_−10%_/°C	*T*_−20%_/°C	*T*_−30%_/°C
1	DMC-Blank	56.84	74.86	157.15	319.21
2	DMC-PF_6_	109.37	237.84	490.36	658.71
3	DMC-BF_4_	190.58	261.45	460.78	627.74
4	DMC-Br	118.30	323.58	522.91	664.67
5	DMC-Cl	106.41	299.95	505.23	708.94

### Analysis of copolymer

**Fig. 5:**
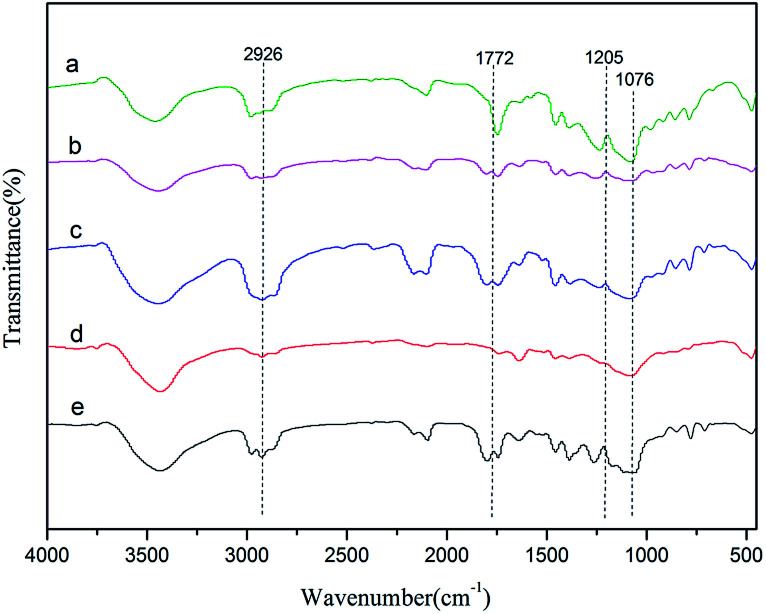
FT-IR spectra of PPC-Blank (a), PPC-PF_6_ (b), PPC-BF_4_ (c), PPC-Br (d) and PPC-Cl (e).

PPC was synthesized by using the Zn–Fe DMC catalysts for the copolymerization of CO_2_ and PO. As shown in Fig. 5, for example, the peak of DMC-PF_6_ at 1772 cm^−1^ can be assigned to the vibration absorption of C=O. The C–O stretching vibration appears at 1205 cm^−1^. These results demonstrate that an ether backbone was formed in the polymers. Furthermore, the bands that appear at 2926 cm^−1^ are attributed to the C–H stretching vibrations. Those at 1076 cm^−1^ were assigned to the C–O–C vibrations. The latter groups were formed as a result of the polymerization of propylene oxide. This confirmed that PPC was successfully obtained by using Zn–Fe DMC catalysts.

**Fig. 6:**
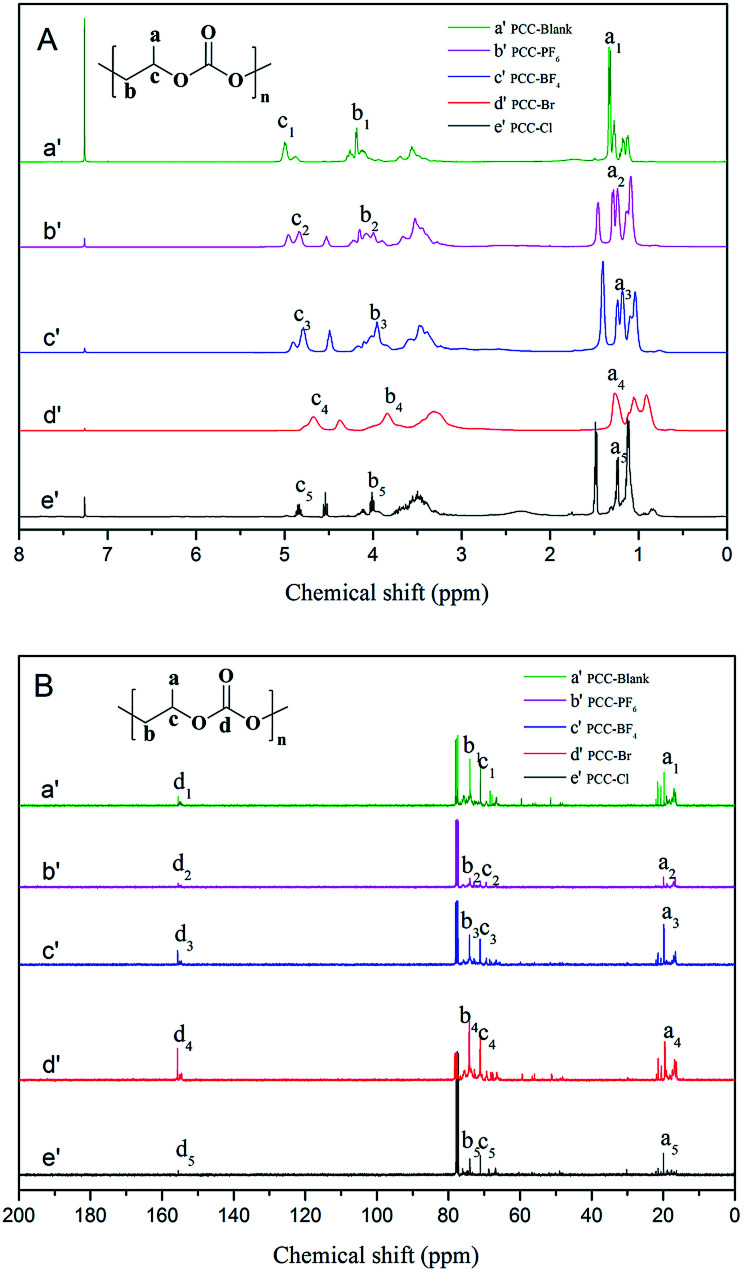
^1^H (A) and ^13^C (B) NMR spectra of PPC-Blank (a′), PPC-PF_6_ (b′), PPC-BF_4_ (c′), PPC-Br (d′) and PPC-Cl (e′).

^1^H and ^13^C NMR spectra of the synthesized PPC samples in CDCl_3_ are shown in Fig. 6(A) and (B), respectively. As evident from Fig. 6(A), three sets of peaks centered at 0.96–1.48 ppm indicate similar H environments in the resultant copolymers, which was assigned to the H of the methyl group. In addition, the two sets of peaks at ∼4.06 ppm are attributed to H having two C–H linkages. The peak at 4.78 ppm indicates the presence of the methylidyne proton. As shown in Fig. 6(B), ^13^C NMR: *δ* (ppm) *a* = 16.5, *b* = 69.3, *c* = 71.4, and *d* = 155.1. This further substantiated the presence of both the carbonate and ether backbone in the resultant copolymers.

**Scheme 2:**
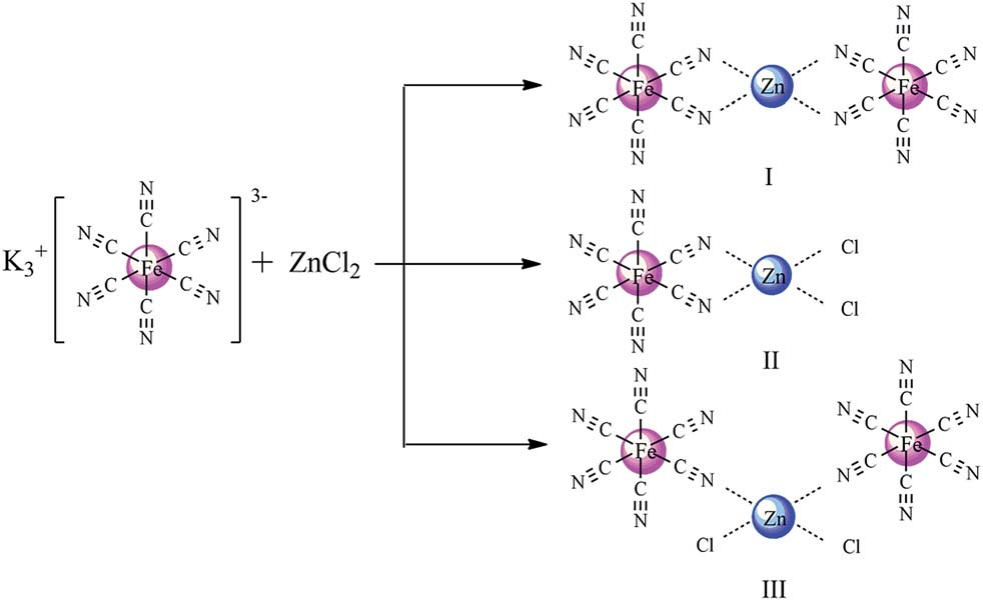
Proposed structures of the Zn–Fe DMC catalysts.

**Fig. 7:**
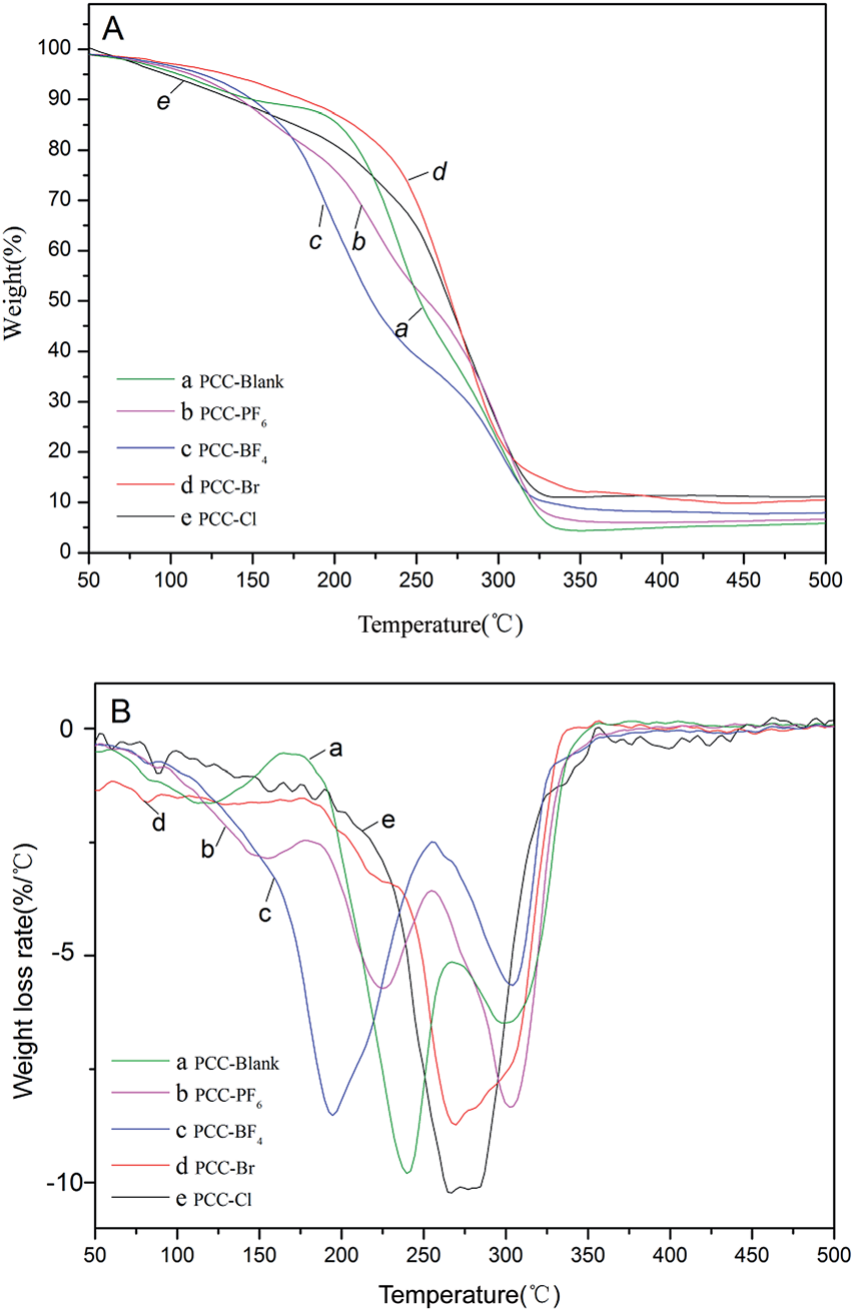
TGA (A) and DTG (B) profiles of PPC-Blank (a), PPC-PF_6_ (b), PPC-BF_4_ (c), PPC-Br (d) and PPC-Cl (e).

Copolymerization of CO_2_ and PO was performed using the same amount of different Zn–Fe DMC catalysts and their catalytic activities are compared (Table 3, entries 1–5). As seen in the table, the DMC-Blank catalyst has the highest number average molecular weight (*M*_n_) (4981) and the lowest content of CO_2_ (18.48%) (Table 3, entry 1). Among the copolymers produced by the different DMC catalysts, DMC-PF_6_ was the most active for the copolymerization of CO_2_ and PO (Table 3, entry 2). The DMC-BF_4_ catalyst had the highest content of CO_2_ (29.00%) and PPC (53.85%). These results indicate that imidazolium-based ionic liquids play an important role in enhancing the activity of Zn–Fe DMC catalysts. Scheme 2 depicts the coordination insertion mechanism. The Zn–Fe DMC catalysts showed high catalytic activity because of the assistance of ILs in terms of strong steric hindrance and the electronic environment. From the obtained results and the previously reported data on the interaction energies between the cations and anions employed in this study, the trend of PF_6_^−^ > BF_4_^−^ > Br^−^ > Cl^−^ is observed. This trend is also apparent based on their catalytic activity.^43^ In particular, PF_6_^−^ was found to be a highly efficient catalyst for this protocol. The weight losses of the acquired PPC are shown in Fig. 7. It was determined that the decomposition temperature of PPC-PF_6_ decreased slightly with an increase in thermal stability.

**Table 3:** Results of PO and CO_2_ copolymerization with ground DMC as catalysts^a^

Entry	Polymer	Catalyst	*M*_n_/*M*_w_/PDI	TON^b^	Composition (%)^c^			TGA_−10%_/°C
					*f*_CO_2__	*f*_PO_	*f*_PC_	
1	PPC-Blank	DMC-Blank	4981/5378/1.08	0.53	18.48	76.98	29.89	149.41
2	PPC-PF_6_	DMC-PF_6_	3354/3377/1.01	4.40	26.81	67.44	48.28	140.79
3	PPC-BF_4_	DMC-BF_4_	2964/2973/1.00	1.20	29.00	65.00	53.85	145.40
4	PPC-Br	DMC-Br	3751/3798/1.01	2.53	26.51	67.78	47.54	177.49
5	PPC-Cl	DMC-Cl	3584/3631/1.01	1.31	24.74	69.30	43.32	136.07

a Reaction conditions: PO 30 mL, the Zn–Fe DMC complexes 0.5 g, CO_2_ pressure 3 MPa, 60 °C 24 h.

b Turnover number(TON) in g·polymer/g·Zn and turnover frequency in g·polymer/g·Zn h.

c Determined by ^1^H NMR spectra. *f*_CO_2__ = (*A*_4.78_ + *A*_4.06_) × 44/(*A*_4.78_ + *A*_4.06_) × 44 + (*A*_4.78_ + *A*_4.06_) × 58 + *A*_3.27–3.73_ × 58. *f*_PO_ = (*A*_4.78_ + *A*_4.06_ + *A*_3.24–3.71_)/[2(*A*_4.78_ + *A*_4.06_) + *A*_3.27–3.73_]. *f*_PC_ = (*A*_4.78_ + *A*_4.06_)/(*A*_4.78_ + *A*_4.06_ + *A*_3.27–3.73_).

### Mechanism

**Scheme 3:**
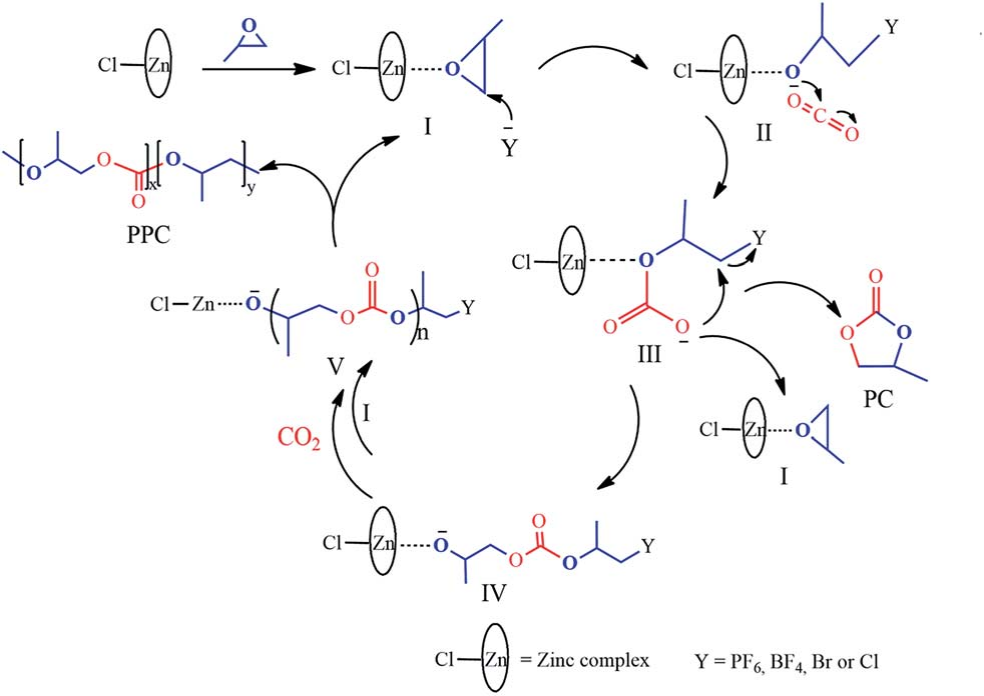
Proposed mechanism of the alternating copolymerization of PO and CO_2_ in the presence of the Zn–Fe DMC catalysts with structure III.

Based on experimental findings, the structures of the Zn–Fe DMC catalysts^29^ and a possible mechanistic pathway of the catalyzed copolymerization reaction are proposed (Scheme 3). The PO monomer initially undergoes ring-opening and is activated by a zinc complex to form the intermediate I.^44^ The synergistic effect of the iron(iii)-cyano-group and the active Zn^2+^ center in the zinc complex facilitated the insertion of activated CO_2_ into PO.^45,46^ We inferred that the nucleophilic anion Y^−^ (PF_6_, BF_4_, Br, or Cl) of the IL attacked the less sterically hindered carbon atom of the activated epoxide to afford intermediate II. Then the oxygen atom of the ring-opening epoxide attacked CO_2_ at the central carbon atom to form intermediate III. Thereby, it transfers to the propagating metal polymer chain *via* intermediate III followed by the facilitation of the activated PO insertion to produce intermediate IV. Cyclic carbonate (PC) was produced accompanied with the loss of Y^−^*via* intramolecular nucleophilic substitution or cyclization of intermediate III.^47,48^ Alternating copolymer polycarbonate V was formed by the further insertion of CO_2_ and the activated PO molecule to ultimately yield the product PPC. The zinc complex and nucleophilic anion Y^−^ of the IL work in cooperation with each other, which is essential to enable intramolecular synergistic action in copolymerization. PPC-PF_6_ exhibited surprisingly high selectivity, likely due to the strong steric hindrance from the coordinated carboxyl groups (when Y^−^ = PF_6_, BF_4_, Br, or Cl in Scheme 3) and the electron atmosphere.

## Conclusions

Zn–Fe DMC catalytic systems were successfully synthesized by the ball milling method, which is beneficial for the formation of more active sites. Imidazolium-based ionic liquids as cocatalysts were introduced into the structure of catalysts. The modified catalysts exhibited higher catalytic activity for the alternating copolymerization of CO_2_ and PO. The Zn–Fe DMC catalysts also exhibited good stability during the catalytic reactions performed at moderately high temperatures. It was also determined that the DMC-PF_6_ catalyst is highly efficient for the copolymerization reaction, while the use of the DMC-BF_4_ catalyst results in a high content of CO_2_ (29.00%) and poly(propylene carbonate) (53.85%).

## Conflicts of interest

There are no conflicts to declare.

## Supplementary Material
